# Effects of an information shock on registry-based health indicators: Evidence from a Swedish PFAS crisis

**DOI:** 10.1371/journal.pone.0340815

**Published:** 2026-01-15

**Authors:** Mikael Elinder, Oscar Erixson, Erik Grönqvist, Sophie Langenskiöld, Mattias Öhman

**Affiliations:** 1 Department of Economics, Uppsala University, Sweden; 2 Centre for Health Economics Research (HEFUU), Uppsala University, Sweden; 3 Institute for Housing and Urban Research, Uppsala University, Sweden; 4 Health Economics, Department of Medical Sciences, Uppsala University, Sweden; UF: University of Florida, UNITED STATES OF AMERICA

## Abstract

This study investigates how the public announcement of very high levels of the toxic chemicals PFAS in the drinking water in Kallinge, Sweden, impacted the health of local residents. By leveraging the unexpected timing of the announcement and utilizing high-frequency individual-level healthcare data, we can isolate the effects of the information shock independently of any toxic effects of PFAS exposure. Despite numerous reports of distress and concern among those affected, our analysis reveals no significant increase in health care utilization following the announcement, as measured by prescription drug use and specialist healthcare visits. This finding holds true even when specifically examining mental health-related care, prescriptions of antidepressants, and sleeping medications. Overall, our results suggest that both the affected community and public authorities managed the situation effectively, mitigating potential health consequences related to anxiety.

## Introduction

Per- and polyfluoroalkyl substances (PFAS) have emerged in recent years as a substantial threat to public health, with potential reproductive effects, increased risk of certain cancers, reduced immune function, and developmental delays in children [1]. However, proving causal links has been challenging, and further research on potential health effects of PFAS exposure is therefore highly needed [2,3].

PFAS refers to a group of more than 10,000 manufactured chemical pollutants that have been used since the early 1940s in the production of non-stick coatings, water-repellent surfaces, and fire-fighting foams [4]. Although many countries have phased out or banned many of these chemicals, they remain a major concern due to their slow degradation and the difficulty of removing them from the environment [5].

While it is now widely recognized that PFAS are essentially everywhere – in drinking water, food, clothing, kitchen appliances, skincare products, and children’s toys – alarming reports of elevated PFAS levels continue to surface. These reports are often linked to leakage from industrial production and fire training sites. Prominent historical examples include Parkersburg (United States), the Veneto Region (Italy), and Düsseldorf (Germany), where leakage from contaminated sites into waterways has made drinking water a significant route of PFAS exposure for hundreds of thousands of people [5].

Primarily, research on PFAS has focused on their direct health consequences, specifically the toxic effects of PFAS exposure, and suggests that the associated social costs related to attributable diseases are likely to be enormous [1]. A growing body of literature has also documented that environmental crises evoke stress, fear and anxiety in the affected society, with negative health effects, in particular related to mental health, that extend beyond those caused by the toxic agents themselves [6–17]. For instance, a recent systematic review found that chronic environmental contamination is associated with elevated levels of anxiety, general stress, depressive symptoms, and PTSD symptoms [17]. Notably, the review also highlighted that individuals, who attribute their health issues to the contamination, appear to be particularly vulnerable to these psychological effects, as well as individuals who felt the government had delegitimised their concerns. However, only a few studies have specifically examined public reaction to information about PFAS contamination [6–9].

While these latter studies, based on interviews and surveys of individuals living in contaminated communities, have found increased anxiety and reduced mental well-being following information about the contamination [6–9] it is important to assess how these self-reported mental health problems manifest in health care utilization to better understand how severe they are and how they translate into illness and formal diagnoses. It should be noted that stress induced by PFAS contamination may not be limited to concerns regarding one’s own health but also to financial stress following, e.g., reduced property prices [6,18].

A growing literature also emphasizes that effective crisis management and communication strategy can mitigate anxiety and related health problems [19–21] following PFAS contamination. It emphasizes that if there is high trust in the governing institutions in the affected community, it facilitates effective management. But once a crisis has occurred, it is suggested to adhere to the following six fundamental principles: Be first, Be right, Be credible, Express empathy, Promote action, Show respect [19–21].

In this paper, we focus on one of the most severe PFAS scandals in the world [22–24] and examine how its announcement and the immediate crisis management impacted various health indicators of the affected individuals. We use various measures of healthcare utilization as indicators of health and study the short-term effects of receiving information about many years of very high PFAS exposure, focusing on the first and second 90-day periods (quarters) after the disclosure on December 16, 2013.

In September–October 2013, the County Administrative Board of Blekinge conducted an expanded groundwater survey that included sampling for various pollutants, including PFAS [25]. Initial results received in November 2013 showed markedly elevated PFAS levels in the Brantafors aquifer, which supplies the small town of Kallinge (approximately 5,000 inhabitants) in Ronneby municipality, southern Sweden. Follow-up tests were conducted to confirm the finding and indicated PFAS concentrations of approximately 10,000 ng/L – exceeding the guideline value at the time (90 ng/L) by more than 100-fold, and surpassing the current guideline value of 4 ng/L by roughly 2,500-fold [22]. Following confirmation, the municipality immediately decided to close the Brantafors water plant and switch the drinking-water supply to a non-contaminated source; this decision was implemented and communicated to the public on 16 December 2013. The contamination received coverage in both national and local media [26]. Media reports noted that the measured levels far exceeded the European Food Safety Authority’s (EFSA) limit for safe intake via drinking water. However, the Swedish Food Agency emphasized that the elevated PFAS levels did not pose an acute health risk [26]. At that time, it was not yet known that the source of the contamination was firefighting foam previously used at the nearby military airbase, nor that the contamination had been ongoing since the mid-1980s [22]. In response to the alarm, municipal officials contacted a research group from Lund University (Occupational and Environmental Medicine) to investigate whether Kallinge residents had elevated PFAS levels in their blood [27]. Findings from a pilot study, published in early March 2014, revealed that children at a school supplied by the affected water plant had several hundred times higher PFAS levels in their blood than children at a school supplied by an unaffected plant [22]. Testing of the overall population of Kallinge, initiated in June 2014, yielded similar results, with the mean PFAS concentration (sum of PFHxS, PFOA, and PFOS) being 234 ng/ml in females and 292 ng/ml in males, compared to 6.0 ng/ml and 7.5 ng/ml, respectively, in a non-exposed population [22,27]. In fact, the blood PFAS levels in Kallinge also exceeded those found in residents of Parkersburg [28] and are referred to as among the highest ever measured [29].

Although knowledge about the health risks associated with PFAS exposure in humans was limited at the time [26], the municipality described the situation as a “catastrophe,” and a leading environmental chemist referred to it as one of the largest chemical disasters in Swedish history in a national newspaper [30]. Studies of the residents in the area have later shown an increased number of kidney cancer cases, elevated cholesterol levels, increased incidence of osteoporosis, and cases of type 2 diabetes [27]. The PFAS contamination in Kallinge has since also become a longstanding legal case concerning responsibility, specifically whether the blame lies with the Swedish military or the municipality responsible for the local water supply company. After several years of trials in various courts, the Supreme Court ultimately ruled that the municipal water company must pay damages to those affected due to their elevated levels of PFAS in their blood. A total of 154 people, including 22 children, are entitled to compensation under the ruling.

While there has been no systematic evaluation of how Kallinge residents reacted to the information shock per se, anecdotal evidence suggests widespread concern [23]. For instance, the local primary care center was inundated with questions about the contamination from anxious residents, and local grocery stores reported a surge in bottled water sales following the announcement [31], indicating that people avoided tap water despite reassurances about its safety. A local group was formed to address residents’ concerns and to advocate for their interests in the law cases that followed. In addition, 3,500 persons, corresponding to 20 percent of the residents in Ronneby, or 70 percent of Kallinge’s residents, enrolled in the blood testing of PFAS administrated by the municipality [27]. These anecdotes suggest that the exposure was perceived as a major health risk at the time, despite considerable uncertainty about the actual risk.

At the same time, the local authorities engaged in active crisis management, largely in line with best practices [20,21], such as shutting down the affected water plant, initiating blood testing, and disseminating information, which can be assumed to have had mitigating effects. It should also be noted that Swedes generally have a high level of trust in authorities and their communication compared to international standards [32,33], with residents in Ronneby not being an exception [34].

In this paper, we aim to investigate whether the reported anxiety caused by the PFAS announcement in Kallinge translated into worse health in terms of increased healthcare utilization. For this purpose, we have collected individual-level administrative data on hospital visits and drug prescriptions, which allows us to examine various forms of healthcare utilization in response to the announcement. We also have data on diagnoses and types of prescribed drugs (e.g., sleeping medication and antidepressants), enabling us to study whether the announcement impacted health.

We assume that any toxic effects of PFAS are not likely to change immediately after the announcement compared to before as it is the level of PFAS accumulating in the bodies of exposed individuals for decades that matters. Moreover, we will assume that if the announcement had substantial effects on the health of affected residents, we would expect these effects to materialize within six months of the announcement. The impact of the announcement, that one’s own drinking water has been toxic, can then be estimated by comparing the health records of individuals who were exposed or not exposed to the announcement. Individuals in carefully selected control areas where the water did not contain elevated levels of PFAS were defined as unexposed.

The results reveal no meaningful effects of the information shock on any of the indicators for the two quarters following the announcement, whether negative or positive. The findings are robust to various specification checks and sensitivity analyses.

The absence of effects on health care utilization suggests that societal and institutional actors effectively addressed reasonable worries and anxiety caused by information of this kind, and that the information was delivered and received in a way that prevented unwarranted negative health effects. This complements previous findings that news of lead contamination in drinking water in Flint (USA) increased primary care visits, including blood lead testing, while reducing emergency department visits [11,35].

## Materials and methods

The research in this paper has been approved by The Swedish Ethical Review Authority, reference number 2022-06739-02. The data were first accessed for the research reported in this study on 22 December 2022. The data are pseudo-anonymized, and the researchers had no access to information that could identify individual participants during or after data collection.

### Empirical strategy

The announcement on December 16, 2013, of high levels of PFAS in the drinking water in Kallinge was unexpected. In a region of fairly similar inhabitants, some received an information shock of being exposed to PFAS, whereas others did not. We exploit the unexpected, i.e., exogenous, nature of the local media reports on December 16, to assess the implications of the shock and the consecutive events that took place during the following two quarters: media coverage and reassurance from the Swedish Food Agency (during the following weeks), information that blood test would be conducted on children (29 January), reports of very high levels of PFAS in children’s blood (26 March), information that all adults are offered blood tests for PFAS (30 May), blood test conducted among adults (2–14 June). Adult test results were reported in the third quarter. Our objective is to estimate the effects of the events in the two first quarters. In case the events result in worse health outcomes for the affected individuals, we capture the impact in terms of register-based health indicators reflecting both health care contacts and use of prescription drugs.

**Fig 1:**
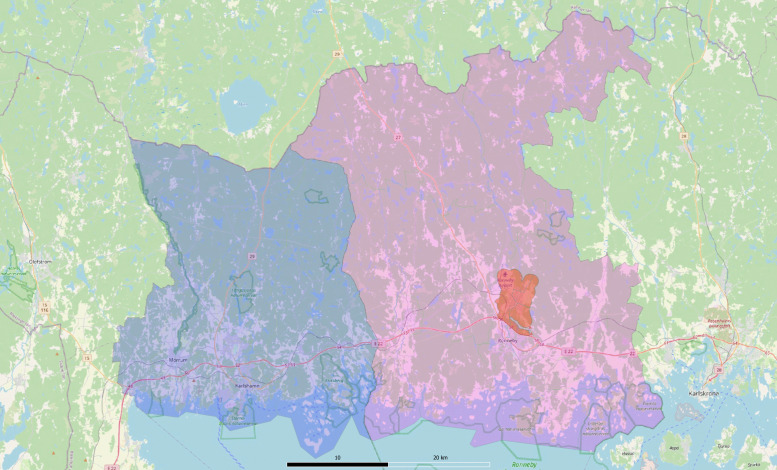
Map of treated and control areas. The orange area corresponds to the exposed area, Kallinge, pink area represents Ronneby municipality, and blue area corresponds to Karlshamn municipality. Contains information from OpenStreetMap and OpenStreetMap Foundation, which is made available under the Open Database License.

The empirical challenge in analyzing the impact of these events is to find a good estimate of the counterfactual outcome. To infer a counterfactual outcome for exposed individuals in Kallinge, we use residents in the whole municipality Ronneby (excluding Kallinge) and residents in the neighboring municipality Karlshamn, as of December 15, as a comparison group in a *difference-in-differences design* using data during the eight quarters before and eight quarters after the initial announcement. In Table 1, we show that residents in comparison areas (Fig 1) are similar to the residents in the exposed area with respect to socioeconomic and demographic background characteristics, validating the choice on comparison group.

**Table 1:** Descriptive statistics for the treatment and control group.

	Treated				Untreated			
	mean	sd	min	max	mean	sd	min	max
Female	0.489	0.500	0	1	0.500	0.500	0	1
Year of birth	1964.96	17.61	1924	1995	1964.38	17.72	1919	1995
Foreign-born	0.109	0.312	0	1	0.091	0.288	0	1
Upper sec. school	0.719	0.450	0	1	0.787	0.410	0	1
Employed	0.593	0.491	0	1	0.622	0.485	0	1
Disp. income (K SEK)	192	105	−187	2,775	214	273	−1,915	28,602
Married	0.453	0.498	0	1	0.460	0.498	0	1
Outpatient (Any)	0.177	0.381	0	1	0.173	0.378	0	1
Drugs (Any)	0.383	0.486	0	1	0.376	0.484	0	1
Drugs (N05-06)	0.094	0.292	0	1	0.076	0.265	0	1
N	3,093				36,863			

*Notes:* Individuals age 18 years or older. All variables are measured in 2013. Binary variables are reported as shares.

In the analysis, we assume that individuals in the contaminated area, due to the information shock, became aware that they had consumed PFAS-contaminated drinking water, while individuals in the comparison group remained unaffected by this information, at least in the dimensions we investigate in this study.

The treatment effect can be calculated by comparing the change in outcomes for exposed individuals before and after the information shock to the change for individuals who were not affected [36]. The difference-in-differences approach leverages the temporal and cross-sectional variation to estimate the causal effect of the exposure (i.e., the information shock) by comparing changes in outcomes over time between a treatment group and a control group. The identifying assumptions for giving the estimates a causal interpretation are, first, that exposed and unexposed individuals would have followed the same trajectory in outcomes in the absence of the information shock (known as the parallel trends assumption); second, that the exposure was unexpected, i.e., that no one knew about the PFAS contamination before December 16 (exogenous treatment); and third, that the treatment is stable, i.e., no interference across treated individuals and consistent treatment of those treated.

The first two assumptions, the parallel trends assumption and the exogenous treatment assumption, are likely to hold in this setting. The third assumption is not likely to hold in the classical sense. Here, this partly works in our favor as our interest lies in the full effect of the information shock and part of the effect stems from how individuals communicate and influence one another. However, if individuals in the control group are also impacted by the shock, this would bias the estimates towards zero. This is not directly testable but would likely manifest as changes in the trajectory of outcomes for the control group after the treatment, which can be investigated.

Given the validity of these assumptions, we can capture short-run effects using a two-ways fixed effects (TWFE) event study specification [36] of the form:


$${\text{Y}}_{\text{it}}=\alpha_{\text{i}}+\alpha_{\text{t}}+\sum_{\tau \neq -\text{1}}\gamma_{\tau }\left[{\text{D}}_{\text{i}}\times \text{1}\left\{\text{t}-\text{S}=\tau \right\}\right]+{\beta X}_{it}+{\text{e}}_{\text{it}}$$ (1)

where *Y*_*it*_ is the outcome for individual *i* in period *t*. Calendar time is indexed by *t* = 1,…,16 corresponding to consecutive 90-day periods aligned to the information shock on December 16, 2013; for ease of interpretation, we label these periods as quarters spanning 2012Q1–2015Q4.

We let *S* denote the quarter containing the information shock, and define event time as *τ = t-S*. Thus, *τ* measures time relative to the information shock: negative values indicate quarters before individuals became aware of the PFAS contamination, while positive values capture quarters after the disclosure.

We define *D*_*i*_* = 1* for individuals residing in the contaminated area of Kallinge, and *D*_*i*_* = 0* otherwise. The interaction term $D_{i}\times \text{1}\left\{t-S=\tau \right\}$ therefore equals one for individuals residing in the contaminated area in event time *τ*, and zero otherwise. The individual fixed effects *α*_*i*_ absorb all time‐invariant differences across individuals, while the period fixed effects *α*_*t*_ capture shocks common to all individuals (e.g., seasonal factors). *X*_*it*_ is a vector of time‐varying controls (education level, employment, disposable income, and marital status).

Each coefficient *γ*_*τ*_ captures the average difference in outcomes between treated and control individuals in event time, and can be interpreted as the average treatment effect on the treated (ATT) in that period. We normalize the coefficients by omitting *τ = −1* (the last quarter before the shock) to avoid perfect multicollinearity, meaning that all estimated effects are relative to that quarter. Our focus is on the treatment effects in the first two post‐shock quarters, subsequently referred to as Q1 (December 16 2013–March 15 2014) and Q2 (March 16 2013–June 13 2014). We estimate Model 1 using a Linear Probability Model (LPM), and cluster the standard errors at the grid of residence level (250 × 250 m) to account for correlation within grids.

### Data

The empirical analysis is based on individual-level panel data from population-wide administrative registers, which we describe below.

The study population is constructed using data from Statistics Sweden and comprises all individuals aged 18 years and older residing in Kallinge, the rest of Ronneby municipality, and the neighboring municipality of Karlshamn as of December 16, 2013. Information about age is obtained from the Register of Total Population [37] and information about place of residence is obtained from the Geography database [38]. In total, the population consists of 39,956 individuals, of whom 3,093 are in the treatment group (Kallinge) and 36,863 in the control group (other parts of Ronneby, and Karlshamn).

For these individuals, we link information, from the Swedish National Board of Health and Welfare, on the date of health care utilization for the period December 27, 2011 to December 5, 2015, which form the basis of our outcome measures (see details below). We aggregate data on the outcomes into an individual-by-quarter panel, where “quarters” are defined as non-overlapping 90-day periods relative to the information shock on December 16, 2013. The first post-shock quarter runs from December 16, 2013 to March 15, 2014, and subsequent quarters advance in 90-day increments; pre-treatment quarters are defined analogously by stepping back in time. This setup yields eight pre-treatment and eight post-treatment quarters.

For each individual, we also link socioeconomic and demographic information, which is available annually.

### Outpatient care

From the Swedish National Patient Register [39], we obtain data on all specialist outpatient visits and ambulatory (emergency department) contacts. This information includes the dates of the visits, allowing us to precisely identify when an individual seeks treatment or care, as well as the diagnoses associated with the visit, captured by ICD10 codes. Outpatient care includes, for example, visits to doctors’ offices or clinics for routine check-ups, treatments, and surgical procedures that do not require an overnight stay. Primary care office visits are not recorded in a centralized national register and thus not part of our outpatient care measure.

Our main outcome is an indicator variable for whether the individual has had any outpatient visit for any reason, hereafter *Outpatient (Any)*.

To investigate mechanisms, we create two additional indicator variables. The first one captures whether the individual has had a visit due to mental health issues, identified by a main diagnosis from ICD chapter F00-99: “Mental and Behavioral Disorders”, hereafter referred *Outpatient (F)*. The second one captures healthcare seeking behavior (e.g., check-ups and screenings) by grouping together outpatient visits with either a main diagnosis from ICD chapter R00–R99: “Symptoms, signs and abnormal clinical and laboratory findings, not elsewhere classified”, or from ICD chapter Z00–Z99: “Factors influencing health status and contact with health services”, hereafter *Outpatient (R or Z)*.

### Drug prescriptions

The Swedish Prescribed Drug Register [40] provides data on all expedited prescription medications, including the dates of prescription, as well as the classification of the drug according to the Anatomical Therapeutic Chemical (ATC) system. These data allow us to capture the use of specific drugs and determine when their usage begins.

We define two outcomes using these data. The first outcome is an indicator variable that takes the value of one if the individual has received any prescribed drug during the quarter, and otherwise zero, hereafter *Drugs (Any)*. The second specifically focuses on drug prescriptions related to mental health and is an indicator of whether the individual has been prescribed any drugs intended to relieve anxiety (e.g., antipsychotics, sedatives, and hypnotics, captured by ATC: N05) or to treat mental health conditions and enhance mood (e.g., antidepressants, captured by ATC: N06), hereafter *Drugs (N05–06).*

### Inpatient care

Inpatient care data, retrieved from the Swedish National Patient Register [39], include information on hospital admissions for conditions serious enough to require treatment or monitoring for an extended period, typically involving at least one overnight stay. Because such hospitalizations are rare, we have limited statistical power for detecting treatment effects. We therefore use these data solely to assess the robustness of our main results. For this purpose, we create an indicator variable for whether the individual has had any hospital admission for any reason, hereafter *Inpatient (Any)*.

### Socioeconomic and demographic characteristics

We collected a set of socioeconomic and demographic variables from the Integrated Database for Labor Market Research (LISA) [41], which combines various administrative registers. These variables are used to assess the validity of the identifying assumptions and to test for heterogeneous responses across different population groups. The variables considered are measured annually, and include: year of birth (used to define age), gender (used to distinguish between men and women), taxable income, education level (used to define high school graduates), marital status (used to distinguish married individuals), country of birth (used to define foreign-born individuals), and employment status (used to identify employed individuals).

## Results

### Descriptive statistics

Table 1 compares the means across the treated and the controls along a number of different predetermined (measured during 2013) demographic and economic characteristics, as well as the main health indicators. We note that there are about equally large shares of women and foreign-born in the two groups, that they – on average – are of similar age and are married to a similar extent. However, the treatment group consists of a lower share of individuals with at least upper secondary education, a lower share of employed individuals, which in turn manifest in a lower average income. Regarding the main outcomes, the two groups have similar levels of outpatient care visits and drug prescriptions. However, the share of drugs prescribed for mental health issues is somewhat higher for the treatment group in the pre-period.

While the descriptive statistics generally suggest that the treatment and control groups are comparable, we assess the robustness of our empirical estimates by estimating models with these individual characteristics as control variables, and estimate heterogeneous effects in the Supporting information.

### Health indicators

**Fig 2:**
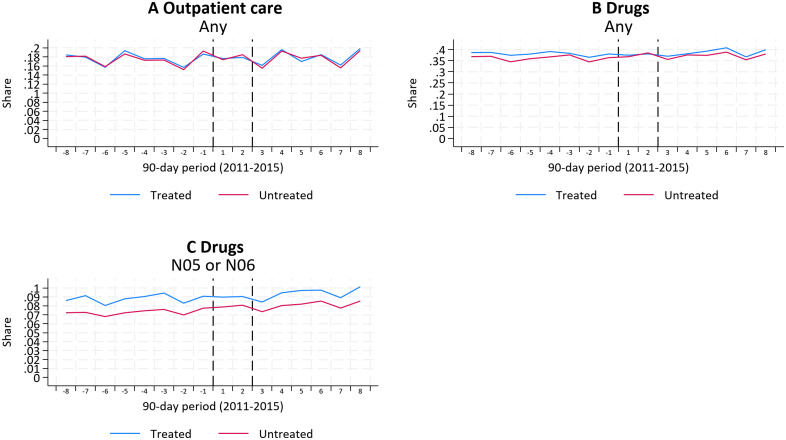
Health indicators by 90-day periods during 2011–2015. Panel A displays outpatient care admissions: share with admissions for any cause. Panel B displays drug prescriptions: share with any prescribed drugs. Panel C displays drug prescriptions: share with prescriptions of psycholeptics and psychoanaleptics. Dashed lines indicate treatment period. Outcomes are described in Data section.

In Fig 2, we provide descriptive evidence of how the main outcomes evolve over time. Considering all three outcomes, we note that they evolve similarly for the treatment and control groups during the eight quarters prior to the information shock. This suggests that the parallel trends assumption is satisfied. In S2 Fig, we assess this more formally, using an event study design, and conclude that no differences in pre-trends are statistically significant. Moreover, S1 Table, presents a formal test of the parallel trends assumption where we test the joint significance of the pre-treatment coefficients which suggest that the assumptions are valid.

Turning to the post period, we observe that the outcomes continue to evolve similarly across the two groups, indicating no discernible impact of the information shock. This pattern is further confirmed by the estimates from our empirical model. Table 2 reports the event study estimates for the first and second quarters following the shock, and the estimates are both statistically insignificant and quantitatively small. For example, for the outcome *Drugs (N05–06)*, we estimate a decline in consumption of about 0.2–0.4 percentage points during the first two quarters – far from statistically significant. To put these estimates into perspective, they represent at most 5 percent of the pre-period baseline mean (8.8 percent). Similar magnitudes are observed for outpatient specialist care and overall drug use, where estimated effects correspond to changes of only a few percent of the pre-period mean. Detailed results for all periods are presented in S2 Table.

**Table 2:** Regression estimates of the impact of the information shock, in the first and second quarter.

	Outpatient	Drugs	
	Any	Any	N05-N06
Q1	0.0092	−0.0098	−0.0023
	(0.0083)	(0.0106)	(0.0053)
Q2	0.0007	−0.0206	−0.0037
	(0.0082)	(0.0110)	(0.0047)
Mean of outcome	0.1762	0.3807	0.0881
R2	0.2703	0.3513	0.4529
N	639,307	639,307	639,307

*Notes:* Estimates are obtained from model, with controls for time-varying individual characteristics, (education, employment and income, and marital status). Variables are described in Data section. Standard errors, clustered at 250 × 250 m grid level, are reported in parentheses. Q1 and Q2 refer to first and second quarters following the announcement of the PFAS contamination (December 16–March 15 and March 16–June 13). Mean of outcome is for the treatment group in the period before the announcement.

### Robustness and sensitivity analyses

In the Supporting Information, we complement our main analysis with a series of robustness and sensitivity checks, which are summarized below.

In S3 Table, we examine whether the average effects mask heterogeneous responses across subgroups by estimating separate models for men and women, and for foreign-born and native individuals. This analysis reveals no meaningful differences across groups.

To assess whether our quarterly estimates obscure important variation in timing or content of information releases, we re-estimate Model 1 at the monthly level. Because the outcomes are rare at the monthly level, moving to a finer temporal resolution reduces precision, which is reflected in the results presented in S4 Table. Although we observe statistically significant increases in one estimate (any drug, first month following the announcement), precision is generally too poor overall to support firm conclusions. Conversely, when aggregating to half-year periods (S5 Table), we gain precision but still find no evidence of increased health care utilization. If anything, we observe indications of reductions in drug prescriptions.

S6 Table evaluates the sensitivity of our results to alternative control groups, motivated by concerns that geographical proximity may generate spillover effects. In the first specification, we use the bordering municipality of Karlshamn as the control group (excluding the non-treated area of Ronneby). In the second specification, we use the non-bordering municipality of Sölvesborg (yet in the same health care region). The resulting estimates are quantitatively similar to our main results. Likewise, our findings are robust to changing the model specification from an LPM to a Logit model; S7 Table reports the corresponding marginal effects.

We also examine a number of alternative outcomes, presented in S2 Table. To study the intensive margin of responses, we analyze the number of outpatient visits and drug prescriptions. These estimates are quantitatively similar to those for the main outcomes and remain statistically insignificant. To assess whether the outpatient visit measure masks responses related to, for example, anxiety or increased demand for general health check-ups, we investigate visits resulting in diagnoses in ICD Chapter F00–99 (“Mental and Behavioral Disorders”) and visits coded as symptoms, signs, or routine examinations (ICD Chapters R00–R99 and Z00–Z99). While these estimates are statistically insignificant, they are somewhat larger in magnitude than our main estimates, likely reflecting the limited precision associated with these relatively rare outcomes. We additionally examine whether the announcement led to increased inpatient care utilization. Given the low incidence of inpatient admissions, these estimates are necessarily imprecise; although small in absolute terms, they do not permit meaningful conclusions.

Finally, S4 Fig provides a descriptive assessment of whether alcohol consumption served as a coping mechanism for stress and anxiety related to the shock [42,43]. Using monthly data on alcohol sales from Systembolaget – the government-owned retail monopoly – we compare sales in the Ronneby store (treated) with those in the Karlshamn store (control). While the two stores followed similar trends prior to the announcement, we find no evidence of a change in alcohol sales in Ronneby thereafter, suggesting that residents did not increase their alcohol consumption in response to the information shock.

Taken together, these robustness and sensitivity checks reinforce our main findings: we find no evidence that the revelation of PFAS exposure led to increased health care utilization, health contingencies, or behaviors commonly associated with stress and anxiety.

## Conclusion

On December 16, 2013, citizens in Kallinge were informed that their drinking water had been contaminated with very high levels of PFAS. At that time, no “safe levels” of PFAS in drinking water had been established, and there was no consensus regarding the health effects of PFAS. While anecdotes and media reports indicate substantial worry among the affected population, we find no evidence that being informed about the high PFAS levels caused any significant health problems. In particular, we observe no effects on the use of prescription drugs or on the utilization of outpatient specialist care.

The availability of high-frequency data enables us to estimate the effects of this information shock without significant confounding by potential toxicological impacts, which occur gradually as PFAS accumulates in the body. Given that the population had been exposed to contaminated water for up to 30 years, there is no reason to expect a sudden onset of toxicological health effects coinciding with the announcement.

Overall, our results suggest that individuals exposed to very high PFAS levels managed the news without experiencing severe worry or anxiety that translated into increased health care utilization. These findings could indicate that the societal and institutional responses were largely appropriate in balancing the delivery of alarming information with the need to avoid inducing excessive anxiety. A Swedish qualitative study suggests, for example, that such coping is facilitated when risk communication is transparent, acknowledges uncertainties, and maintains an open and proactive dialogue with the public [44]. Our results relate to previous studies documenting self-reported deterioration in mental health in response to PFAS contamination [6–9], as well as to evidence from Flint (USA), where information about lead contamination increased primary care visits and reduced emergency room visits [11].

Our outcomes are not directly comparable to the primary care (office visit) margin studied in Flint, because primary care visits are not observed in national registers in Sweden. Danagoulian et al. [11] show that the Flint advisory increased office visits and lead testing while avoidable emergency department use fell, indicating substitution toward primary care after the information shock. In contrast, we observe no short-run changes in specialist outpatient care, ED/ambulatory care, or drug use. This suggests that if any increase in primary care reassurance visits occurred in Kallinge – as local reports indicate – we do not find evidence that it translated into medication initiation or escalation to specialist or emergency care. However, it should be noted that the absence of detectable effects does not rule out small effects (which we cannot detect with statistical precision) or responses along other margins not captured in our outcomes.

What factors contributed to the apparently successful communication regarding the PFAS contamination and its potential health effects? First, several public authorities – including primary care providers, the municipality, the National Food Agency, and others – actively took responsibility for managing the crisis. The municipality’s offer of widespread PFAS blood testing may also have reduced uncertainty and thereby lowered the likelihood that reassurance-seeking escalated beyond primary care. Second, the local community organized effectively, holding multiple information meetings for concerned residents. This, combined with the high levels of trust Swedes generally have in public institutions [33] – and particularly in the Public Health Agency of Sweden, the country’s most trusted public institution – likely mitigated the impact of the information shock.

External validity likely depends on institutional and health system context. Our findings are most informative for high-trust settings with universal health care coverage and rapid local crisis management, where public uncertainty may be resolved quickly within primary care and through organized testing. In lower-trust settings, or where access barriers push worried residents toward emergency care, information shocks may yield different utilization patterns, as observed in Flint [11].

## Supporting information

S1 Fig Health indicators by 90-day periods during 2011–2015. Panel A displays outpatient care admissions: share with admissions for mental and behavioral disorders (F). Panel B displays outpatient care admissions: share with admissions for symptoms not elsewhere classified and health status reasons for encounters with health services (R or Z). Panel C displays inpatient care admissions: any cause. Dashed lines indicate treatment period. Outcomes are described in Data section.
(TIF)

S2 Fig Event study plots: Panel A displays outpatient care admissions: share with admissions for any cause. Panel B displays drug prescriptions: share with any prescribed drugs. Panel C displays drug prescriptions: share with prescriptions of psycholeptics and psychoanaleptics. Estimates are obtained from Model 1, with controls for time-varying individual characteristics (education, employment and income, and marital status). Standard errors are clustered at the 250 × 250 meter grid level. Blue vertical lines indicate 95% confidence intervals. The dashed lines represent the average estimates across the pre-, treatment-, and post-periods.
(TIF)

S3 Fig Distributional plot for count measures: Panel A displays the quarterly number of outpatient care admissions for any cause, year 2013. Panel B displays the quarterly number of drug prescriptions, year 2013.
(TIF)

S4 Fig Alcohol sales in Ronneby and Karlshamn by month during 2012–2015. Dashed lines indicate treatment period. Sales are measured in liters of pure alcohol.
(TIF)

S1 Table Test of parallel pre-trends. Test of joint significance (*F*-test) for the pre-period estimates obtained from model 1.
(RTF)

S2 Table Regression estimates (quarterly level). The estimates are obtained from model 1 with controls for time-varying individual characteristics, (education, employment and income, and marital status). Standard errors clustered at 250 × 250 m grid level, are reported in parentheses. Outcomes are described in the Data section in the main text. S3 Fig provides distribution plots for the count variables. Mean of outcome is for the treatment group in the period before the announcement. ^*^ indicates a *p*-value below 0.05.
(RTF)

S3 Table Heterogeneous effects (quarterly level). The estimates are obtained from model 1 with controls for time-varying individual characteristics, (education, employment and income, and marital status). Standard errors clustered at 250 × 250 m grid level, are reported in parentheses. Outcomes are described in the Data section in the main text. Mean of outcome is for the treatment group in the period before the announcement. ^*^ indicates a *p*-value below 0.05.
(RTF)

S4 Table Regression estimates (monthly level). The estimates are obtained from model 1 with controls for time-varying individual characteristics, (education, employment and income, and marital status). Standard errors clustered at 250 × 250 m grid level, are reported in parentheses. Outcomes are described in the Data section in the main text. Mean of outcome is for the treatment group in the period before the announcement. ^*^ indicates a *p*-value below 0.05.
(RTF)

S5 Table Regression estimates (half-year level). The estimates are obtained from model 1 with controls for time-varying individual characteristics, (education, employment and income, and marital status). Standard errors clustered at 250 × 250 m grid level, are reported in parentheses. Outcomes are described in the Data section in the main text. Mean of outcome is for the treatment group in the period before the announcement. ^*^ indicates a *p*-value below 0.05.
(RTF)

S6 Table Alternative control group (quarterly level). The estimates are obtained from model 1 with controls for time-varying individual characteristics, (education, employment and income, and marital status). Standard errors clustered at 250 × 250 m grid level, are reported in parentheses. Outcomes are described in the Data section in the main text. Mean of outcome is for the treatment group in the period before the announcement. In the left panel, Kallinge (treated) is compared with Karlshamn (control). In the right panel, Kallinge (treated) is compared with the non-bordering municipality Sölvesborg (control).
(RTF)

S7 Table Marginal effect estimates with logit specifications (quarterly level). Marginal effects estimates are obtained from a Logit specification of model 1 with controls for time-varying individual characteristics, (education, employment and income, and marital status). Due to computational complexity standard errors are non-clustered, with fixed effects for group and quarter. Outcomes are described in Data section in the main text. ^*^ indicates a *p*-value below 0.05.
(RTF)
