# Supplementary material for: Effects of an information shock on registry-based health indicators: Evidence from a Swedish PFAS crisis
Source: PLoS One. 2026 Jan 15;21(1):e0340815. doi: 10.1371/journal.pone.0340815 (PMC12806844; doi:10.1371/journal.pone.0340815)
Supplement: S1 Table — Test of joint significance (F-test) for the pre-period estimates obtained from model 1. (RTF) [file pone.0340815.s005.rtf]

	F-value	p-value	
Main outcomes	
Outpat. (Any)	0.4371	0.8793	
Drugs (Any)	0.8919	0.5118	
Drugs (N05-N06)	0.4141	0.8941	
Additional outcomes	
Outpat. (Any, count)	1.5252	0.1541	
Outpat. (F)	1.3576	0.2192	
Outpat. (R or Z)	1.5320	0.1519	
Drugs (Any, count)	2.1304	0.0376	
Inpat. (Any)	0.5440	0.8015	
N = 639,307
