# Supplementary material for: Effects of an information shock on registry-based health indicators: Evidence from a Swedish PFAS crisis
Source: PLoS One. 2026 Jan 15;21(1):e0340815. doi: 10.1371/journal.pone.0340815 (PMC12806844; doi:10.1371/journal.pone.0340815)
Supplement: S2 Table — The estimates are obtained from model 1 with controls for time-varying individual characteristics, (education, employment and income, and marital status). Standard errors clustered at 250 × 250 m grid level, are reported in parentheses. Outcomes are described in the Data section in the main text. S3 Fig provides distribution plots for the count variables. Mean of outcome is for the treatment group in the period before the announcement. * indicates a p-value below 0.05. (RTF) [file pone.0340815.s006.rtf]

Regressions with controls (quarterly)
		Outpatient		Drugs		Inpatient	
		Any	Any, count		F	R or Z		Any	N05-N06	Any, count		Any	
-8		0.0105	-0.0052		-0.0039	0.0029		0.0021	0.0004	0.0004		0.0030	
		(0.0104)	(0.0193)		(0.0029)	(0.0066)		(0.0113)	(0.0049)	(0.0206)		(0.0044)	
-7		0.0046	-0.0144		-0.0039	0.0087		0.0010	0.0054	0.0430		0.0011	
		(0.0083)	(0.0182)		(0.0034)	(0.0050)		(0.0113)	(0.0056)	(0.0229)		(0.0047)	
-6		0.0054	-0.0086		-0.0052*	0.0079		0.0127	-0.0010	0.0652*		0.0045	
		(0.0071)	(0.0172)		(0.0025)	(0.0052)		(0.0115)	(0.0053)	(0.0238)		(0.0044)	
-5		0.0139	0.0283		-0.0016	0.0159*		0.0043	0.0023	0.0398		0.0002	
		(0.0090)	(0.0198)		(0.0034)	(0.0059)		(0.0094)	(0.0048)	(0.0209)		(0.0041)	
-4		0.0100	-0.0042		-0.0029	0.0080		0.0075	0.0026	0.0309		0.0001	
		(0.0086)	(0.0170)		(0.0026)	(0.0046)		(0.0110)	(0.0056)	(0.0253)		(0.0046)	
-3		0.0102	-0.0010		-0.0041	0.0076		-0.0094	0.0050	-0.0114		0.0031	
		(0.0071)	(0.0179)		(0.0028)	(0.0047)		(0.0112)	(0.0051)	(0.0188)		(0.0037)	
-2		0.0117	0.0090		-0.0003	0.0098		0.0040	-0.0002	0.0288		0.0012	
		(0.0093)	(0.0156)		(0.0031)	(0.0055)		(0.0115)	(0.0049)	(0.0201)		(0.0042)	
-1 (Ref.)													
													
1		0.0092	0.0104		-0.0028	0.0054		-0.0098	-0.0023	0.0139		-0.0036	
		(0.0083)	(0.0137)		(0.0029)	(0.0040)		(0.0106)	(0.0053)	(0.0223)		(0.0039)	
2		0.0007	-0.0241		-0.0032	0.0073		-0.0206	-0.0037	-0.0257		0.0063	
		(0.0082)	(0.0183)		(0.0027)	(0.0050)		(0.0110)	(0.0047)	(0.0220)		(0.0044)	
3		0.0130	-0.0018		-0.0043	0.0081		-0.0023	-0.0025	-0.0098		0.0021	
		(0.0079)	(0.0157)		(0.0031)	(0.0043)		(0.0110)	(0.0049)	(0.0205)		(0.0040)	
4		0.0098	0.0061		-0.0002	0.0021		-0.0113	0.0010	-0.0220		0.0048	
		(0.0091)	(0.0223)		(0.0026)	(0.0051)		(0.0100)	(0.0053)	(0.0189)		(0.0053)	
5		-0.0005	-0.0078		-0.0008	0.0098		0.0026	0.0019	0.0001		-0.0017	
		(0.0086)	(0.0173)		(0.0022)	(0.0051)		(0.0106)	(0.0055)	(0.0203)		(0.0044)	
6		0.0082	-0.0090		0.0035	0.0095		0.0031	-0.0011	0.0115		0.0016	
		(0.0088)	(0.0205)		(0.0026)	(0.0051)		(0.0113)	(0.0062)	(0.0233)		(0.0050)	
7		0.0131	0.0221		0.0002	0.0079		-0.0034	-0.0017	-0.0045		0.0047	
		(0.0091)	(0.0178)		(0.0028)	(0.0052)		(0.0109)	(0.0058)	(0.0219)		(0.0038)	
8		0.0112	0.0036		-0.0041	0.0135*		0.0029	0.0027	-0.0026		0.0028	
		(0.0100)	(0.0188)		(0.0026)	(0.0049)		(0.0105)	(0.0054)	(0.0225)		(0.0040)	
Upper sec. school		-0.0120*	-0.0218*		-0.0008	-0.0008		-0.0336*	-0.0047	-0.0556*		0.0003	
		(0.0051)	(0.0094)		(0.0022)	(0.0028)		(0.0060)	(0.0031)	(0.0113)		(0.0017)	
Employed		0.0017	-0.0021		-0.0020*	0.0038*		-0.0001	-0.0020	-0.0034		-0.0004	
		(0.0026)	(0.0056)		(0.0009)	(0.0016)		(0.0027)	(0.0016)	(0.0068)		(0.0010)	
Income		-0.0000	-0.0000		-0.0000	-0.0000		0.0000	0.0000	-0.0000		-0.0000	
		(0.0000)	(0.0000)		(0.0000)	(0.0000)		(0.0000)	(0.0000)	(0.0000)		(0.0000)	
Married		-0.0032	-0.0094		-0.0017	-0.0009		-0.0121*	-0.0094*	-0.0164		-0.0012	
		(0.0046)	(0.0093)		(0.0014)	(0.0030)		(0.0046)	(0.0028)	(0.0120)		(0.0018)	
Constant		0.1861*	0.2992*		0.0186*	0.0527*		0.4001*	0.0869*	0.6943*		0.0253*	
		(0.0045)	(0.0087)		(0.0019)	(0.0026)		(0.0057)	(0.0027)	(0.0110)		(0.0016)	
Mean of outcome		0.1762	0.2732		0.0149	0.0540		0.3807	0.0881	0.6819		0.0279	
R2		0.2703	0.3074		0.4071	0.1724		0.3513	0.4529	0.4737		0.1300	
N		639,307	639,307		639,307	639,307		639,307	639,307	639,307		639,307	
