# Supplementary material for: Effects of an information shock on registry-based health indicators: Evidence from a Swedish PFAS crisis
Source: PLoS One. 2026 Jan 15;21(1):e0340815. doi: 10.1371/journal.pone.0340815 (PMC12806844; doi:10.1371/journal.pone.0340815)
Supplement: S3 Table — The estimates are obtained from model 1 with controls for time-varying individual characteristics, (education, employment and income, and marital status). Standard errors clustered at 250 × 250 m grid level, are reported in parentheses. Outcomes are described in the Data section in the main text. Mean of outcome is for the treatment group in the period before the announcement. * indicates a p-value below 0.05. (RTF) [file pone.0340815.s007.rtf]

Regressions with controls (quarterly), heterogenitity
	Outpatient (Any)		Drugs (Any)		Drugs (N05-06)	
	Men	Women	Native	Foreign-born		Men	Women	Native	Foreign-born		Men	Women	Native	Foreign-born	
Q1	0.0171	0.0009	0.0103	0.0007		-0.0085	-0.0110	-0.0091	-0.0148		-0.0007	-0.0039	-0.0014	-0.0089	
	(0.0127)	(0.0115)	(0.0091)	(0.0263)		(0.0109)	(0.0182)	(0.0105)	(0.0347)		(0.0060)	(0.0093)	(0.0058)	(0.0227)	
Q2	0.0134	-0.0127	-0.0003	0.0107		-0.0210	-0.0202	-0.0226*	-0.0040		0.0033	-0.0108	-0.0022	-0.0148	
	(0.0141)	(0.0107)	(0.0095)	(0.0263)		(0.0128)	(0.0153)	(0.0111)	(0.0327)		(0.0065)	(0.0069)	(0.0052)	(0.0177)	
Mean of outcome	0.1487	0.2050	0.1757	0.1808		0.3086	0.4561	0.3818	0.3720		0.0714	0.1056	0.0878	0.0906	
R2	0.2700	0.2636	0.2718	0.2561		0.3633	0.3147	0.3511	0.3508		0.4538	0.4466	0.4553	0.4247	
N	320,462	318,845	580,003	59,304		320,462	318,845	580,003	59,304		320,462	318,845	580,003	59,304	
