# Supplementary material for: Effects of an information shock on registry-based health indicators: Evidence from a Swedish PFAS crisis
Source: PLoS One. 2026 Jan 15;21(1):e0340815. doi: 10.1371/journal.pone.0340815 (PMC12806844; doi:10.1371/journal.pone.0340815)
Supplement: S4 Table — The estimates are obtained from model 1 with controls for time-varying individual characteristics, (education, employment and income, and marital status). Standard errors clustered at 250 × 250 m grid level, are reported in parentheses. Outcomes are described in the Data section in the main text. Mean of outcome is for the treatment group in the period before the announcement. * indicates a p-value below 0.05. (RTF) [file pone.0340815.s008.rtf]

Regressions with controls (monthly)
	Outpatient		Drugs	
	Any		Any	N05-N06	
-24	0.0003		0.0028	0.0046	
	(0.0068)		(0.0087)	(0.0041)	
-23	0.0007		0.0021	0.0045	
	(0.0060)		(0.0098)	(0.0043)	
-22	0.0097		0.0108	0.0043	
	(0.0068)		(0.0070)	(0.0039)	
-21	0.0016		0.0103	0.0073	
	(0.0065)		(0.0093)	(0.0040)	
-20	0.0018		0.0138	0.0082	
	(0.0063)		(0.0087)	(0.0042)	
-19	0.0000		0.0156	0.0084	
	(0.0073)		(0.0092)	(0.0054)	
-18	0.0017		0.0111	0.0029	
	(0.0058)		(0.0089)	(0.0038)	
-17	-0.0052		0.0124	0.0059	
	(0.0073)		(0.0089)	(0.0039)	
-16	0.0050		0.0231*	0.0073	
	(0.0057)		(0.0086)	(0.0044)	
-15	0.0153*		0.0075	0.0085	
	(0.0070)		(0.0070)	(0.0044)	
-14	0.0091		0.0120	0.0017	
	(0.0062)		(0.0088)	(0.0037)	
-13	0.0054		0.0129	0.0048	
	(0.0069)		(0.0095)	(0.0050)	
-12	-0.0007		0.0099	0.0072	
	(0.0059)		(0.0089)	(0.0044)	
-11	0.0031		0.0175	0.0087	
	(0.0073)		(0.0091)	(0.0048)	
-10	0.0041		0.0134	0.0059	
	(0.0068)		(0.0092)	(0.0048)	
-9	0.0007		0.0016	0.0080	
	(0.0061)		(0.0073)	(0.0044)	
-8	0.0043		0.0004	0.0045	
	(0.0076)		(0.0088)	(0.0040)	
-7	0.0027		0.0049	0.0054	
	(0.0061)		(0.0083)	(0.0043)	
-6	0.0090		0.0173*	0.0048	
	(0.0060)		(0.0081)	(0.0038)	
-5	0.0040		0.0070	0.0052	
	(0.0064)		(0.0087)	(0.0039)	
-4	-0.0009		0.0055	0.0078	
	(0.0072)		(0.0078)	(0.0051)	
-3	0.0046		0.0107	0.0065	
	(0.0062)		(0.0085)	(0.0043)	
-2	0.0012		0.0146	0.0091*	
	(0.0083)		(0.0084)	(0.0045)	
-1 (Ref.)					
					
1	0.0051		0.0166*	0.0060	
	(0.0052)		(0.0081)	(0.0041)	
2	0.0035		-0.0049	0.0058	
	(0.0060)		(0.0098)	(0.0041)	
3	0.0051		-0.0013	0.0020	
	(0.0058)		(0.0092)	(0.0038)	
4	-0.0024		0.0021	0.0034	
	(0.0065)		(0.0101)	(0.0050)	
5	-0.0029		-0.0063	0.0073	
	(0.0061)		(0.0081)	(0.0037)	
6	0.0014		-0.0050	0.0027	
	(0.0068)		(0.0094)	(0.0034)	
7	0.0096		0.0112	0.0085*	
	(0.0060)		(0.0093)	(0.0039)	
8	0.0052		0.0106	0.0079*	
	(0.0065)		(0.0085)	(0.0039)	
9	0.0023		-0.0052	-0.0007	
	(0.0056)		(0.0089)	(0.0041)	
10	0.0032		-0.0040	0.0033	
	(0.0072)		(0.0075)	(0.0042)	
11	0.0022		0.0176*	0.0074	
	(0.0065)		(0.0071)	(0.0043)	
12	0.0054		-0.0093	0.0057	
	(0.0073)		(0.0084)	(0.0047)	
13	0.0071		0.0002	0.0015	
	(0.0064)		(0.0069)	(0.0039)	
14	0.0045		0.0045	0.0081*	
	(0.0066)		(0.0097)	(0.0040)	
15	-0.0058		0.0196*	0.0100*	
	(0.0061)		(0.0096)	(0.0045)	
16	0.0025		0.0069	0.0026	
	(0.0089)		(0.0102)	(0.0044)	
17	0.0060		0.0177	0.0066	
	(0.0074)		(0.0095)	(0.0045)	
18	0.0011		0.0090	0.0013	
	(0.0067)		(0.0091)	(0.0045)	
19	0.0066		-0.0038	0.0040	
	(0.0068)		(0.0083)	(0.0045)	
20	0.0088		0.0185*	0.0109*	
	(0.0053)		(0.0088)	(0.0052)	
21	0.0078		0.0016	-0.0011	
	(0.0066)		(0.0092)	(0.0037)	
22	0.0034		0.0071	0.0086	
	(0.0073)		(0.0092)	(0.0052)	
23	0.0030		-0.0004	0.0083	
	(0.0083)		(0.0086)	(0.0043)	
24	0.0060		0.0062	0.0024	
	(0.0071)		(0.0086)	(0.0042)	
Upper sec. school	-0.0069*		-0.0141*	-0.0009	
	(0.0025)		(0.0030)	(0.0017)	
Employed	-0.0002		-0.0024	-0.0022*	
	(0.0012)		(0.0015)	(0.0008)	
Income	-0.0000*		-0.0000	-0.0000	
	(0.0000)		(0.0000)	(0.0000)	
Married	-0.0026		-0.0056*	-0.0051*	
	(0.0023)		(0.0026)	(0.0014)	
Constant	0.0829*		0.1851*	0.0371*	
	(0.0023)		(0.0028)	(0.0014)	
Mean of outcome	0.0752		0.1784	0.0378	
R2	0.1400		0.1995	0.2390	
N	1,917,931		1,917,931	1,917,931	
