# Supplementary material for: Effects of an information shock on registry-based health indicators: Evidence from a Swedish PFAS crisis
Source: PLoS One. 2026 Jan 15;21(1):e0340815. doi: 10.1371/journal.pone.0340815 (PMC12806844; doi:10.1371/journal.pone.0340815)
Supplement: S6 Table — The estimates are obtained from model 1 with controls for time-varying individual characteristics, (education, employment and income, and marital status). Standard errors clustered at 250 × 250 m grid level, are reported in parentheses. Outcomes are described in the Data section in the main text. Mean of outcome is for the treatment group in the period before the announcement. In the left panel, Kallinge (treated) is compared with Karlshamn (control). In the right panel, Kallinge (treated) is compared with the non-bordering municipality Sölvesborg (control). (RTF) [file pone.0340815.s010.rtf]

Regressions with controls (quarterly), alternative control group
	Karlshamn		Sölvesborg	
	Outpatient
(Any)	Drugs
(Any)	Drugs
(N05-06)		Outpatient
(Any)	Drugs
(Any)	Drugs
(N05-06)	
Q1	0.0117	-0.0097	-0.0021		0.0127	-0.0024	-0.0022	
	(0.0086)	(0.0109)	(0.0055)		(0.0089)	(0.0113)	(0.0056)	
Q2	0.0039	-0.0187	-0.0040		-0.0028	-0.0152	-0.0031	
	(0.0085)	(0.0114)	(0.0049)		(0.0089)	(0.0117)	(0.0052)	
Mean of outcome	0.1762	0.3807	0.0881		0.1762	0.3807	0.0881	
R2	0.2734	0.3520	0.4619		0.2682	0.3694	0.5151	
N	388,372	388,372	388,372		232,624	232,624	232,624	
