# Supplementary material for: Effects of an information shock on registry-based health indicators: Evidence from a Swedish PFAS crisis
Source: PLoS One. 2026 Jan 15;21(1):e0340815. doi: 10.1371/journal.pone.0340815 (PMC12806844; doi:10.1371/journal.pone.0340815)
Supplement: S7 Table — Marginal effects estimates are obtained from a Logit specification of model 1 with controls for time-varying individual characteristics, (education, employment and income, and marital status). Due to computational complexity standard errors are non-clustered, with fixed effects for group and quarter. Outcomes are described in Data section in the main text. * indicates a p-value below 0.05. (RTF) [file pone.0340815.s011.rtf]

Logit marginal effects with controls (quarterly)
	Outpatient		Drugs		
	Any		Any	N05-N06		
-8	0.0101		0.0018	0.0008		
	(0.0087)		(0.0108)	(0.0049)		
-7	0.0040		0.0004	0.0053		
	(0.0087)		(0.0108)	(0.0049)		
-6	0.0047		0.0124	0.0001		
	(0.0090)		(0.0108)	(0.0050)		
-5	0.0129		0.0038	0.0027		
	(0.0086)		(0.0108)	(0.0049)		
-4	0.0094		0.0070	0.0027		
	(0.0088)		(0.0108)	(0.0049)		
-3	0.0099		-0.0095	0.0044		
	(0.0088)		(0.0108)	(0.0049)		
-2	0.0118		0.0041	0.0005		
	(0.0090)		(0.0108)	(0.0050)		
-1 (Ref.)						
						
1	0.0088		-0.0100	-0.0021		
	(0.0088)		(0.0108)	(0.0049)		
2	0.0001		-0.0210	-0.0036		
	(0.0087)		(0.0108)	(0.0049)		
3	0.0128		-0.0026	-0.0022		
	(0.0089)		(0.0108)	(0.0050)		
4	0.0089		-0.0116	0.0004		
	(0.0086)		(0.0108)	(0.0049)		
5	-0.0013		0.0025	0.0011		
	(0.0088)		(0.0108)	(0.0048)		
6	0.0069		0.0025	-0.0018		
	(0.0087)		(0.0107)	(0.0048)		
7	0.0122		-0.0043	-0.0018		
	(0.0089)		(0.0108)	(0.0049)		
8	0.0096		0.0022	0.0013		
	(0.0086)		(0.0108)	(0.0048)		
Upper sec. school	-0.0202*		-0.0523*	-0.0167*		
	(0.0022)		(0.0030)	(0.0016)		
Employed	-0.0375*		-0.0625*	-0.0201*		
	(0.0016)		(0.0021)	(0.0010)		
Disp. income (K SEK)	-0.0000*		-0.0000	-0.0000*		
	(0.0000)		(0.0000)	(0.0000)		
Married	0.0125*		0.0404*	-0.0091*		
	(0.0017)		(0.0024)	(0.0012)		
N	639,307		639,307	639,307		
